# SAD phasing of XFEL data depends critically on the error model

**DOI:** 10.1107/S2059798319012877

**Published:** 2019-10-30

**Authors:** Aaron S. Brewster, Asmit Bhowmick, Robert Bolotovsky, Derek Mendez, Petrus H. Zwart, Nicholas K. Sauter

**Affiliations:** a Molecular Biophysics and Integrated Bioimaging Division, Lawrence Berkeley National Laboratory, Berkeley, CA 94720, USA; b Center for Advanced Mathematics for Energy Research Applications, Lawrence Berkeley National Laboratory, Berkeley, CA 94720, USA

**Keywords:** XFELs, SAD phasing, error modeling, *cctbx.xfel*, serial crystallography

## Abstract

A nonlinear least-squares method for refining a parametric expression describing the estimated errors of reflection intensities in serial crystallographic (SX) data is presented. This approach, which is similar to that used in the rotation method of crystallographic data collection at synchrotrons, propagates error estimates from photon-counting statistics to the merged data. Here, it is demonstrated that the application of this approach to SX data provides better SAD phasing ability, enabling the autobuilding of a protein structure that had previously failed to be built. Estimating the error in the merged reflection intensities requires the understanding and propagation of all of the sources of error arising from the measurements. One type of error, which is well understood, is the counting error introduced when the detector counts X-ray photons. Thus, if other types of random errors (such as readout noise) as well as uncertainties in systematic corrections (such as from X-ray attenuation) are completely understood, they can be propagated along with the counting error, as appropriate. In practice, most software packages propagate as much error as they know how to model and then include error-adjustment terms that scale the error estimates until they explain the variance among the measurements. If this is performed carefully, then during SAD phasing likelihood-based approaches can make optimal use of these error estimates, increasing the chance of a successful structure solution. In serial crystallography, SAD phasing has remained challenging, with the few examples of *de novo* protein structure solution each requiring many thousands of diffraction patterns. Here, the effects of different methods of treating the error estimates are estimated and it is shown that using a parametric approach that includes terms proportional to the known experimental uncertainty, the reflection intensity and the squared reflection intensity to improve the error estimates can allow SAD phasing even from weak zinc anomalous signal.

## Abstract 2

SAD phasing of XFEL data is shown to require accurate error estimates from the merged reflection intensities. Various methods of treating the errors are presented, including a best-practice approach that refines error-correction terms using a nonlinear least-squares method.

## 1. Introduction   

Solving a novel protein structure using X-ray crystallography typically involves either a reliance on a similar structure from which molecular replacement (MR) can be used to derive phasing information, or the presence of heavy atoms that can provide anomalous differences for use in SAD (single-wavelength anomalous dispersion) or MAD (multiple-wavelength anomalous dispersion) phasing (among other methods). In SAD phasing, X-ray anomalous scattering by heavy atoms in the protein structure breaks inversion/Friedel symmetry in the diffraction pattern, with otherwise equivalent reflections typically exhibiting 3–4% differences in intensity. This information can be used to determine the heavy-atom substructure in the protein, which is then used to solve the phasing problem. This approach requires highly accurately measured intensities, and the analysis of such data has been shown to benefit from maximum-likelihood methods (de La Fortelle & Bricogne, 1997 ▸; McCoy *et al.*, 2004 ▸), with the caveat that maximum-likelihood methods also require accurate estimates of the merged intensity errors.

In serial crystallography (SX), determining the reflection intensities with the required accuracy and estimating their error is challenging, which has made phasing new structures from SX data difficult. Typically, 10^2^–10^7^ crystals are exposed to either synchrotron or X-ray free-electron laser (XFEL) radiation. Each crystal is exposed once in a random orientation using liquid-stream injection, grid-based raster scanning or acoustic droplet injection (reviewed in Bergmann *et al.*, 2017 ▸). Individual diffraction patterns are indexed to determine the crystal orientation and unit-cell dimensions, and reflection locations are then predicted and integrated. Because the crystals are not rotated the reflections are only partially recorded, and therefore a post-refinement algorithm is used to apply a partiality correction factor in order to re-express the summed intensity in terms of the structure-factor equivalent. Finally, the redundantly measured reflections are merged together using either a simple average or a weighted average (White, 2014 ▸; Kabsch, 2014 ▸; Sauter, 2015 ▸; Uervirojnangkoorn *et al.*, 2015 ▸; Ginn *et al.*, 2015 ▸).

In crystallographic experiments, the error estimates from photon-counting statistics alone do not explain the variance observed in the measurements, always underestimating the variance owing to the presence of other sources of error. In 1985, an IUCr subcommittee on statistical descriptors was tasked to evaluate the validity of the statistical approaches used at the time to determine variances and provide recommendations (Schwarzenbach *et al.*, 1989 ▸). In their report, they suggested that if the multiplicity of the measurements was high enough then simply the spread of the measurements is sufficient to estimate the error. Otherwise, they recommended that crystallographic methods developers use error-propagation approaches to combine uncertainty in photon counting with random and systematic sources of error. Random error sources include readout noise and dark current. Systematic sources of error include X-ray attenuation from air, sample or water, detector misalignment and errors in estimating the wavelength or flux and, in the case of SX, the partiality. Because the reflections are only partially recorded, every measurement is reduced by anywhere from 0% to 100% of its full intensity, depending on the crystal orientation, mosaicity and the spectral characteristics of the beam. It is likely that partiality is the dominant source of error for SX data, where reflection tails touching the Ewald sphere introduce orders of magnitude more uncertainty than reflections that directly intersect the Ewald sphere.

The full list of sources of error is extensive and it is difficult to ensure that all sources of error have been accounted for. To this end, procedures have been developed to adjust error estimates, usually inflating them to larger values, using intensity-dependent and intensity-independent factors, after applying any other known corrections (Leslie, 1999 ▸, 2006 ▸; Otwinowski & Minor, 2001 ▸; Kabsch, 2010*a* ▸,*b* ▸; Evans, 2006 ▸, 2011 ▸). For a full set of references, see Rossmann & Arnold (2001 ▸).

In the present study, we have found that how the error estimates are obtained directly affects our ability to use SAD phasing to solve an XFEL structure *de novo*. We examined three methods for treating error and show that only some of them allowed us to find the Zn sites of a thermolysin data set using SAD and subsequently autobuild the structure. We also show that with better error treatment, interpretable maps can be obtained even with fewer measurements.

## 2. Methods   

This work follows directly from the work reported in Brewster *et al.* (2018 ▸). The data set can be downloaded from cxi.db entry 81 (https://www.cxidb.org/id-81.html), and after indexing and integration consists of over 160 000 crystals from a thermolysin data set collected at the CXI endstation of LCLS on a CSPAD detector (Kern *et al.*, 2014 ▸; Hart *et al.*, 2012 ▸). After indexing, time-dependent ensemble refinement was applied, in which the data were grouped into batches of images and the detector models were then refined to account for the time-dependent shifts in sample position that are likely to arise from instability in the liquid-jetting system (Brewster *et al.*, 2018 ▸). The expected Bijvoet ratio for this system (〈|*F*_+_ − *F*_−_|〉/〈*F*〉), comprising two Zn^2+^ and four Ca^2+^ atoms in a total of 2561 non-H atoms, is 2.1% (Terwilliger *et al.*, 2016 ▸; Hendrickson & Teeter, 1981 ▸).

Unlike in Brewster *et al.* (2018 ▸), the images were first converted from measured pixel values to photon units, dividing them by an estimated value of 25, as reported by the beamline staff. This experiment used an early-generation CSPAD with a non-uniform gain response; therefore, using a single gain-correction constant greatly oversimplifies the physics of the detector (Hart *et al.*, 2012 ▸). With these gain-corrected pixel values, we also needed to modify the merging protocol described in Brewster *et al.* (2018 ▸). We apply a per-image resolution filter during merging, in which the resolution cutoff of each image is determined by the point at which the signal-to-noise ratio (*I*/σ) falls below a given threshold. To compensate for the fact that *I*/σ decreases with the square root of the gain, we decreased the threshold from 0.5 to 0.1 [0.1 = 0.5/(25)^1/2^].

We analyzed three methods for the treatment of error from SX data, as described in Sections 2.1–2.3. After the integrated intensity error estimates had been treated using one of these methods, we used them to create merged intensities *I_h_* and merged error estimates σ*_h_* according to the following procedure. Given a Miller index *h* with *n* measurements of the intensity of *h*, we define the *j*th measurement of *h* as *I* ^P^ *_hj_* and the associated photon-counting error as σ^P^ *_hj_* [referred to as σ_c_(*I_hj_*) in Brewster *et al.* (2018 ▸)]. The superscript P means that the reflection is only partially observed owing to the measurement being from a still image. The intensity and estimated error are both scaled to their full equivalent values, *I_hj_* and σ*_hj_*, using a per-image scale factor *G*_*c*_, a Wilson *B* factor *B*_*c*_ and a per-reflection partiality correction *P_hj_*, all of which were determined during scaling and post-refinement according to Sauter (2015 ▸),
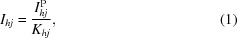


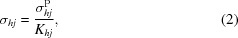


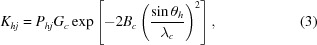
where θ_*h*_ is the Bragg angle for Miller index *h*, λ_*c*_ is the incident wavelength and the subscript *c* denotes the crystal which gave rise to reflection *hj*. *P_hj_* is the partiality-correction factor for this measurement [see equation (14) of Uervirojnang­koorn *et al.* (2015 ▸)], which depends on λ_*c*_, mosaicity estimates and the unit-cell dimensions and orientation of crystal *c*. Importantly, the post-refinement of Sauter (2015 ▸) is similar to the post-refinement described in Winkler *et al.* (1979 ▸) and Rossmann *et al.* (1979 ▸) in that the target function refines the difference between the observed and predicted intensity values. However, the choice of the parameters being refined differs. Here, we refine the misorientation angles of the crystals, *G_c_*, and *B_c_* for each frame, but not the mosaicity itself, which is instead derived from empirically examining which reflections are observed on the image (Sauter *et al.*, 2014 ▸).

After frame-by-frame post-refinement, scaling and partiality correction, we merge the corrected intensities and error estimates according the three protocols detailed below and summarized in Table 1 ▸.

### 2.1. Protocol 1: unweighted mean   

We begin with the suggestion of Schwarzenbach *et al.* (1989 ▸), in which we use the mean of the measurements to estimate the reflection intensity, 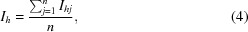
and we use the observed spread of the measurements to determine the error estimates,
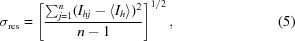


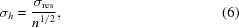
where σ_res_ refers to the residual differences between the measurements and their mean (*i.e.* the standard deviation), and σ_*h*_ refers to the merged error estimate for reflection *h* and is the standard error of the mean. Protocol 1 does not use the information in original error estimates from photon counting (σ_*hj*_), and assumes that a large enough sample of the reflections is available to reliably estimate the uncertainty. This formulation is similar to that in Chapman *et al.* (2011 ▸) and White *et al.* (2012 ▸), differing slightly in the denominator of σ_res_ by using *n* − 1 instead of *n*.

### 2.2. Protocol 2: weighted mean   

The distribution of measurement intensities from still images does not follow a Gaussian distribution because every intensity is measured only partially. The reflection partiality is a function of crystal orientation, unit-cell dimensions, wavelength spectrum and crystal mosaicity. Difficulty in estimating these parameters results in integrating weak and highly partial reflections that skew the distribution towards zero. Because of the skewed distribution, the mean is not an ideal estimator of the structure-factor intensity, and so protocol 2 uses a weighted mean and a weighted standard error of the mean to estimate the reflection intensity and the uncertainty in that estimation,
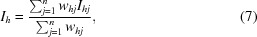


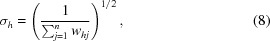
where the weights *w_hj_* are variance weights derived from the photon-counting error estimates σ*_hj_*, *i.e.* the estimated error derived from summing photons as described in Leslie (1999 ▸), which should follow a Poisson distribution:
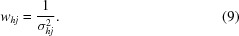



### 2.3. Protocol 3: Ev11   

Protocol 3 adjusts the error estimates using terms from Evans (2006 ▸) and Evans (2011 ▸): *s*_fac_, *s*_B_ and *s*_add_.^1^ In Brewster *et al.* (2018 ▸) we showed that applying these factors to the non-gain-corrected thermolysin data brings down the final merged *I*/σ estimate to around 30, which is more reasonable for protein crystallography (Diederichs, 2010 ▸). We also showed that applying these factors greatly increased the anomalous peak height of the Zn atom (from 44.6σ to 74.0σ). In Brewster *et al.* (2018 ▸), following the example of Evans (2011 ▸), our implementation used a simplex minimizer to refine these terms. In this work, we instead used a gradient-based nonlinear least-squares minimization procedure.

The equation to inflate the estimated error of the individual measurements is
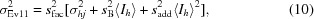
where 〈*I_h_*〉 is the mean of the measurements of *h* after correcting by the factor *K_hj_*. This equation is similar to error propagation, in which additional errors proportional to the intensity, likely derived from instrument instability (*s*_add_), are added in quadrature to the counting-error estimates σ_*hj*_. In Evans (2011 ▸), the *s*_fac_ term is considered to account for effects such as errors in the gain, converting detector counts to photon counts. The *s*_B_ term was included to better fit the observed error estimates to a normal distribution, but in Evans (2011 ▸) the term was given no physical meaning. Here, we first show how we compute initial estimates of *s*_fac_, *s*_B_ and *s*_add_ using normal probability analysis, following Evans (2006 ▸). We then use a limited-memory Broyden–Fletcher–Goldfarb–Shanno (LBFGS; Liu & Nocedal, 1989 ▸) minimizer to refine these parameters until the deviation of normalized error estimates best approaches 1.

After refinement of the *s*_fac_, *s*_B_ and *s*_add_ terms, 1/σ^2^_Ev11_ is used as a weight in (7) and (8) to compute the weighted mean and weighted standard error of the mean of each reflection, as in protocol 2.

#### 2.3.1. Initial parameter estimates   

Estimates of error such as σ_*hj*_ represent the deviation of the measurements *I*_*hj*_ from the unknown population mean value. If these deviations from the mean are normally distributed then the normalized deviations will follow a standard normal distribution, *i.e.* a Gaussian distribution centered on zero with a standard deviation of 1. We choose initial values of *s*_fac_, *s*_B_ and *s*_add_ that best adjust the original deviations such that the normalized deviations approach a standard normal distribution, according to the following procedure.

*Normalized deviations*. This formulation of the normalized deviations of a set of intensities and sigmas is similar to that described in Evans (2011 ▸), but includes the (*n* − 1)/*n* factor as currently implemented by *AIMLESS*. The normalized deviation δ_*hj*norm_ for *I*_*hj*_ is
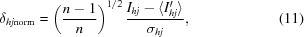
where 〈*I*′*_hj_*〉 is the mean of the measurements of *h* except for *I* *_hj_*. In the special case where *n* = 1, 〈*I*′*_hj_*〉 = 0, and since in that case *n* − 1 = 0, δ^2^_*hj*norm_ = 0. These observations are not included in the normal probability analysis below for the initial parameter estimates.

**Figure 1:**
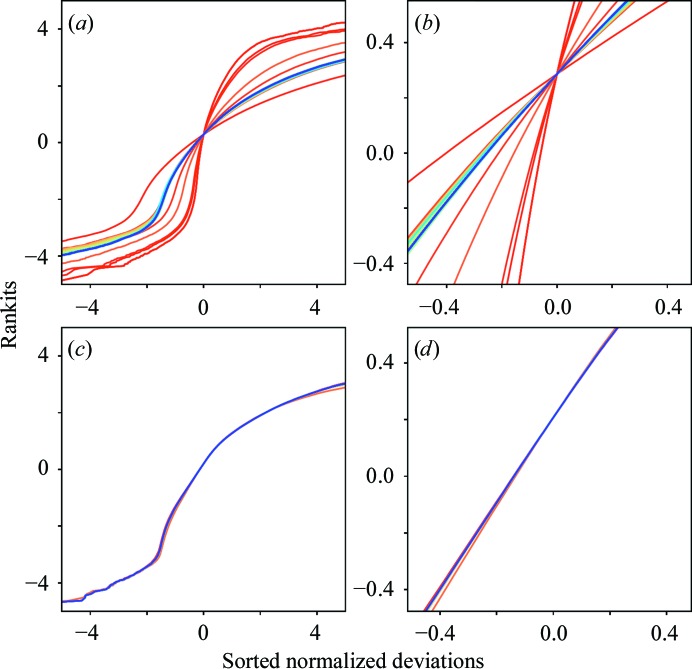
Normal probability plots for 5000 images. A 5000-image subset of the data was merged using protocol 3. During each step of the parameter refinement, a normal probability plot was generated (*a*). The rankits (equation 12) are plotted versus the sorted normalized deviations from the mean (equation 11). Each line represents one step during refinement and is colored using a rainbow color map from red (early steps) to blue (late steps). This is a non-gain-corrected data set. (*b*) Enlargement of the central area of (*a*) used to compute the slope and offset for initialization of the parameters. (*c*) As (*a*) but with a gain-corrected data set, in which each pixel was divided by 25. (*d*) As (*b*) for the central area of (*c*).

*Normal probability analysis*. Using normalized deviations, we can initialize the *s*_fac_, *s*_B_ and *s*_add_ parameters using a graphical technique called a ‘normal probability plot’, as suggested by Evans (2006 ▸) (see also Chambers *et al.*, 1983 ▸). A normal probability plot helps to determine how near a sampling of data approaches a normal distribution. Given a sampling of *m* observations, we sort them and then plot them versus a set of *m* theoretical or expected values. The theoretical values are perfectly distributed according to the normal distribution. If our observations are indeed normally distributed then the plot will be a straight line with slope 1 and offset 0. The ‘perfect’ theoretical values are normal order statistic medians, also referred to as rankits. In the simple case of *m* = 5 total observations, the second, third and fourth rankits are equal to the first quartile, median and third quartile of a normal distribution. We compute the rankits in the same way as *qqnorm* does in *R* (R Core Team, 2017 ▸). The rankit *z*_*i*_ for the *i*th value in *m* is
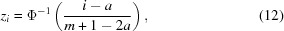
where Φ^−1^ is the standard normal quantile function (the inverse of the cumulative distribution function) and where *a* = 3/8 if *m* ≤ 10 and 0.5 if *m* > 10. The expression (*i* − *a*)/(*m* + 1 − 2*a*) in (12) converts *i* to a number between 0 and 1; therefore, *z*_*i*_ is the expected value of the ranked *i*th sample from a normal distribution. Again, the normal probability plot, or the plot of the rankits versus δ_*hj*norm_ (where all δ_*hj*norm_ are first sorted by value), will have a slope of 1 with an offset of 0 if the error estimates are normally distributed. To determine an initial set of parameters, we determine the slope and offset of a line fitted to the central area of this plot (using the area between −0.5 and 0.5 to avoid fitting outliers). *s*_fac_ is initialized to the slope, as is performed in Evans (2006 ▸). In Evans (2006 ▸), *s*_add_ is set to 0.02. As we did not know whether this value was applicable to XFEL data, we experimented with initializing *s*_add_ to the normal probability plot offset and *s*_B_ to *s*_add_^1/2^. This seemed to give reasonable results. As refinement proceeds, the normal probability plot becomes more linear and the slope approaches 1 as the parameters better correct the estimated errors to approach those derived from sampling a normal distribution (Fig. 1 ▸). Note that the normal probability analysis is only used to initialize the parameters; the refinement of the parameters is outlined below.

### 2.3.2. Parameter refinement   

We refine the *s*_fac_, *s*_B_ and *s*_add_ parameters using the LBFGS quasi-Newton minimizer requiring only first derivatives. For each step, we evaluate (10) for each σ_*hj*_ and then compute the normalized deviations using (11). The target function *f*_σ_ minimizes the deviation of the root-mean-squared deviation (r.m.s.d.) of the normalized deviations from 1, as determined over 100 intensity bins. We bin the intensities as follows. For each Miller index *h*, determine the mean intensity 〈*I*_*h*_〉 of the measurements of *h*. The bin width will be the maximum of all 〈*I*_*h*_〉 for all *h* minus the minimum 〈*I*_*h*_〉 for all *h* divided by 100. For each *h*, all the measurements of *h* will be assigned to a single bin based on 〈*I*_*h*_〉. There will be *m*_*b*_ measurements in intensity bin *b*. Call all the measurements in bin *b* *I*_*bk*_, where *k* ranges from *k* = 1 to *m*_*b*_. Each *I*_*bk*_ is associated with a normalized deviation, δ_*bk*norm_, computed using the adjusted error estimate for that measurement of *h*, 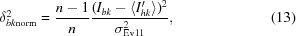
where 〈*I*′_*hk*_〉 is the mean of all measurements of Miller index *h* except for *I*_*bk*_. Here, σ_Ev11_ is the corrected error estimate for measurement *I*_*bk*_ using (10) (note that the subscripts *b* and *k* are suppressed in this reference to σ_Ev11_). The target function is then
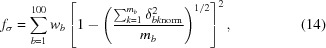
where *b* iterates over the 100 intensity bins. The term for each bin is weighted by *w_b_* = *m_b_* ^1/2^. After refinement of the *s*_fac_, *s*_B_ and *s*_add_ parameters, we apply them to each σ_*hj*_ to compute the final estimated error for each measurement, σ_Ev11_.

The derivatives of the target function (14) with respect to the parameters are shown in Appendix *A* [App appa]. The refinement of these terms using LBFGS is protocol 3.

## 3. Results   

We reprocessed the data files from cxi.db entry 81 (Brewster *et al.*, 2018 ▸) comprising 160 000 lattices, including a gain correction (division of the pixel values by 25) prior to integration, and merged them using *cxi.merge*. In Brewster *et al.* (2018 ▸) the initial scale factors were derived from the known structure of thermolysin. Here, in contrast, we wanted to solve the structure *de novo*, so we used an alternate merging protocol. We first averaged all of the data without post-refinement using the *cxi.merge* default of weighted means and weighted standard errors of the mean (protocol 2). We then used this averaged data set as a scaling reference and merged again, applying post-refinement to each frame, refining the misorientation angles of the crystal, the scale factor and a Wilson *B* factor (Sauter, 2015 ▸), but again using the *cxi.merge* default of weighted means and weighted standard errors of the mean (protocol 2). We then re-merged a third time, using this post-refined data set as a reference for scaling. During this third merging, each of the three error models were applied. This bootstrapping approach to obtain a reference from the unscaled data is similar to how we have merged data before without a reference (Uervirojnangkoorn *et al.*, 2015 ▸). For protocol 3, the final values after refinement were *s*_fac_ = 1.32, *s*_B_ = 0.71 and *s*_add_ = 0.51.

As mentioned above, we applied a gain correction to all images prior to integration, dividing the pixel values by 25 to convert to units of photons. As expected, correcting for gain also had a dramatic effect on the refinement of the SDFAC parameters for Ev11 (protocol 3). We processed a 5000-image subset without gain correction and found that refinement of the SDFAC parameters drove the functional (14) from 3306 to 122, driving the parameters from *s*_fac_ = 7.47, *s*_B_ = 0.72 and *s*_add_ = 0.52 to *s*_fac_ = 4.14, *s*_B_ = 0.00 and *s*_add_ = 0.52 over 66 steps. However, for the gain-corrected data, the refinement drove the functional from 156 to 149, driving the parameters from *s*_fac_ = 1.44, *s*_B_ = 0.67 and *s*_add_ = 0.45 to *s*_fac_ = 1.43, *s*_B_ = 0.96 and *s*_add_ = 0.45 over 14 steps. The difference between the two refinements can be seen in Fig. 1 ▸. Not only did a more substantial minimization need to be performed on the non-gain-corrected data, but the final *s*_fac_ parameter is quite a bit larger in magnitude, indicating a compensation for the absence of a gain correction. It is also worth noting that the difference between the two initial *s*_fac_ values is related to the gain ratio (7.47^2^/1.44^2^ = 26.9), again indicating the relationship between *s*_fac_ and the uncertainty in the gain estimate.

**Figure 2:**
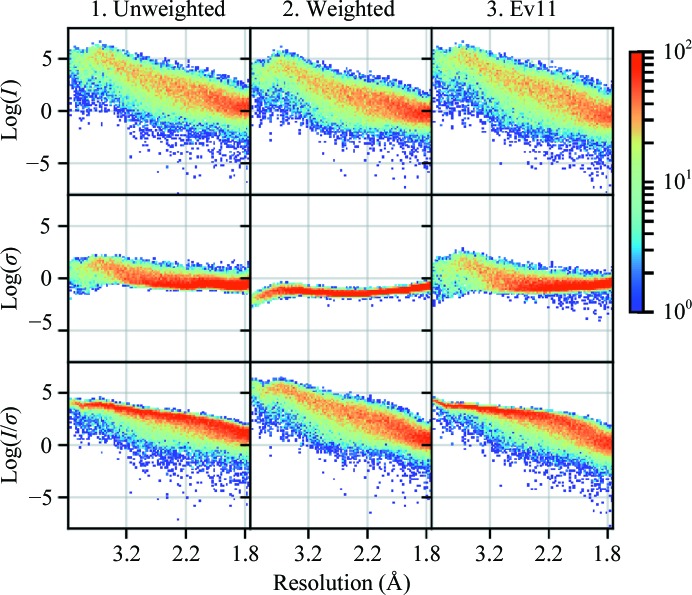
Intensity and σ versus resolution. 2D histograms of *I*, σ and *I*/σ (top, middle and bottom) versus resolution for the three error models. Data are for merged values. Note that the *y* axes and the color are on a logarithmic scale.

**Figure 3:**
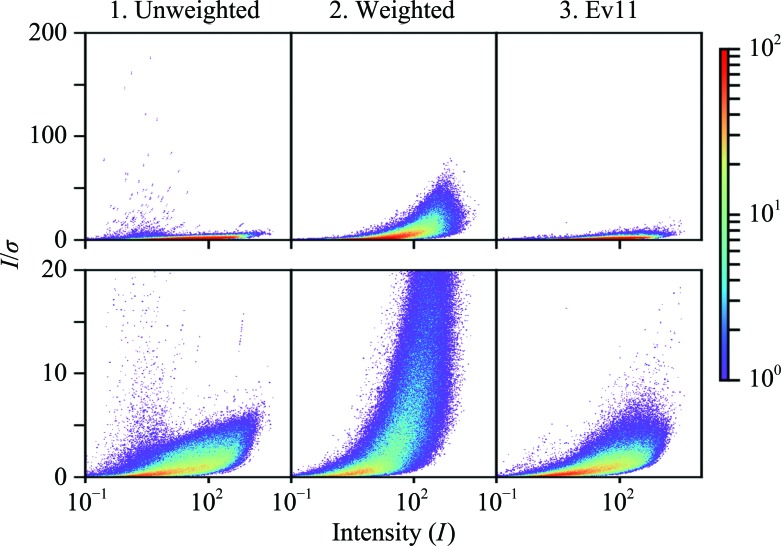
*I*/σ versus *I* plots with different error models. 2D histograms of *I*/σ versus *I* for the three error models. Unmerged intensities and error estimates are shown. In the top and bottom plots the same data are presented but with different scales for the *y* axis. Note that the color is on a logarithmic scale.

Properly scaled, partiality-corrected and merged intensities reported in units of photons from XFELs should be comparable to the full reflection intensities measured at synchrotrons if all systematic effects have been accounted for. One measure of comparison for the two techniques is the signal to noise, or the *I*/σ ratio. Fig. 2 ▸ shows *I*, σ and *I*/σ versus resolution plots for the merged data, which show that the error estimates for protocol 2 are orders of magnitude lower than for protocols 1 and 3. Fig. 3 ▸ shows *I*/σ versus *I* plots for all three data sets, as presented in Diederichs (2010 ▸) (note that these are for the unmerged data). While Diederichs (2010 ▸) was working with reflections that were much better measured and had much lower redundancy, *I*/σ in photons should be comparable (between 20–40), and indeed we see the overall *I* values are of the order that is expected (10^0^–10^4^). Protocols 1 and 3 show *I*/σ values of the order that would be expected from protein crystallography, while protocol 2 has *I*/σ values that are higher than expected. We also see that data sets 1 and 3 do not display the sigmoidal shape demonstrated in Diederichs (2010 ▸), indicating that the signal-to-noise ratio has not reached its limit for this system. This implies there is further work to be performed to remove systematic errors. Finally, note that the scattered data points in protocol 1 (upper left of Fig. 3 ▸) that have high *I*/σ but low *I* come from reflections with low redundancy (≤2–4). These error estimates, which for protocol 1 come only from the standard error of the mean of the observations, become unreliable with low redundancy. It is likely that a redundancy of at least 5 is required for SX data to be reliable using protocol 1.

**Figure 4:**
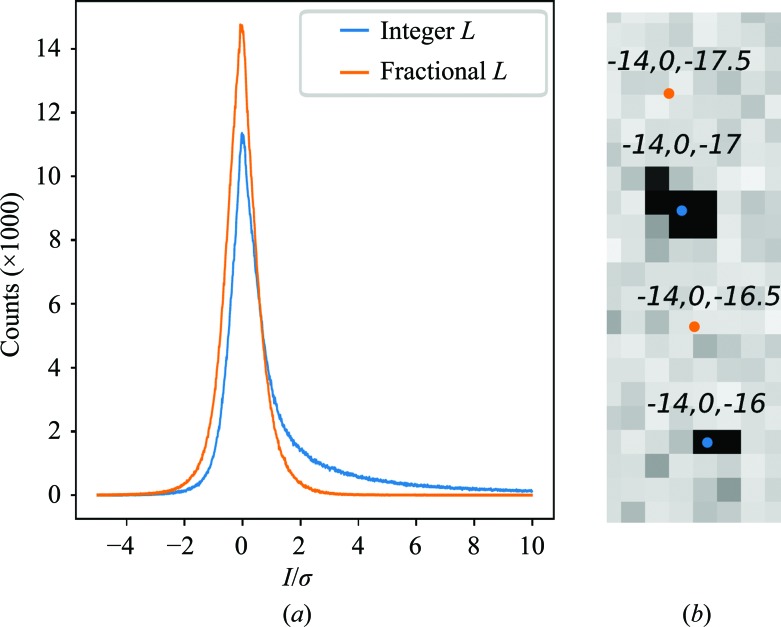
Histogram of *I*/σ for signal versus noise. (*a*) A random subset of 3800 images from one processing run of thermolysin was re-integrated, including the prediction of non-existent reflections at the halfway positions along the c* axis. These predictions, which are halfway between observed reflections, are composed of only noise. (*b*) Example of reflections labeled with integer *L* and fractional *L* indices.

We also examined the overall *I*/σ trends in the data set. In Hattne *et al.* (2014 ▸), we observed numerous intensities at large negative multiples of *I*/σ, and we used these negative measurements to compute an additional error-adjustment term to account for this extra uncertainty. To determine whether this approach (termed Ha14; see also Brewster *et al.*, 2018 ▸) was applicable to the data in this work, we examined the distribution of *I*/σ in a subset of images. We selected integration regions void of Bragg spots and compared the distribution of *I*/σ between these empty measurements and the measurements where signal is predicted. We found that the large negative intensity outliers seen in Hattne *et al.* (2014 ▸) are absent from our data and that the negative intensities have a similar distribution to the empty measurements (see Fig. 4 ▸). Therefore, Ha14 methods do not seem to apply.

Phasing and autobuilding was performed using *phenix.autosol* (Adams *et al.*, 2010 ▸), supplying the thermolysin amino-acid sequence from PDB entry 4tnl (Kern *et al.*, 2014 ▸), one NCS copy and using all defaults, except for specifying two Zn atoms as the search target, using a thorough *HySS* search to 4.0 Å and using a solvent fraction of 0.467 with extreme density modification. Phasing results are shown in Table 2 ▸.

While all protocols were able to find the six heavy-atom sites, protocol 2 essentially failed during SAD phasing and autobuilding, while the unweighted protocol 1 partially succeeded. Over two thirds of the structure was built with protocol 1 and it is likely that the model could be finished manually. Phasing and autobuilding were successful using error estimates inflated by SDFAC parameters (protocol 3). This protocol also showed an improved ability to phase and autobuild the structure compared with using the unweighted variance (protocol 1). The LBFGS version of SDFAC refinement shows nearly the same results as a simplex minimizer (not shown), but importantly LBFGS is deterministic, does not rely on the randomness in the initialization inherent to simplex minimization and converges in less time and in fewer steps than the simplex minimizer (see below).

**Figure 5:**
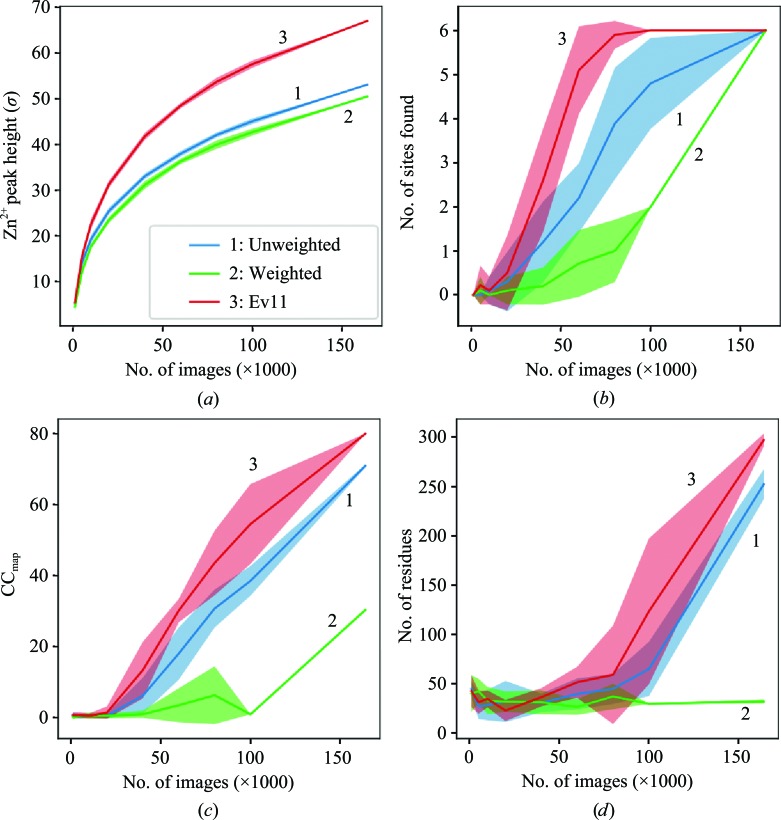
Effect of image count on autobuilding success. For each of the three protocols, increasing numbers of images were processed. The anomalous peak height for the Zn^2+^ atom (*a*), the number of heavy-atom sites found (out of six) (*b*), the known model-to-map CC (*c*) and the number of residues built (*d*) are shown versus the number of images in the data set. In each case, shaded areas indicate the standard deviation of either the ten subsamples (data sets 1000–100 000) or the ten random seeds (full data set, 164 063 images). Note that for (*b*) certain data points have the same number of sites found in all trials and hence have no standard deviation.

To determine whether these algorithms improve the number of images needed for phasing, for each of the three protocols we re-merged the data using increasing numbers of images from 1000 images to the full data set (160 000+ images; Fig. 5 ▸). In addition, since we used random sampling to create these subsets, we repeated this sampling ten times for each subset. For the full data set, which could not be sub-sampled, we instead ran the auto-solver ten times with random seeds, as recommended by Bunkóczi *et al.* (2015 ▸).

We found that for this zinc SAD phasing experiment we still needed nearly all of the images to autobuild the structure. Autobuilding built about half of the structure with 100 000 images with protocol 3 (Fig. 5*d*), but failed with fewer images and with the other protocols. However, we can still examine the phasing ability of the data by examining the Zn^2+^ anomalous peak height (Fig. 5 ▸ *a*), the CC_map_ statistic, which is the correlation of the phased map with the known structure with PDB code 1lnd (Holland *et al.*, 1995 ▸) (Fig. 5 ▸ *c*), and the number of sites found by *phenix.hyss* out of the six possible, as determined by using *phenix.emma* to match sites between the known structure and the SAD-determined sites (Fig. 5 ▸ *b*). SDFAC treatment improves the result over the residual-only treatment (compare protocols 1 and 3). Protocol 2 consistently underperformed.

Finally, a note on the performance of simplex versus LBFGS. Using derivative-based minimization drives the optimization to a similar solution using fewer steps. During one trial (not shown) with 10 000 images, the simplex refiner took 88 steps over 932.8 s. However, the LBFGS minimizer took 51 steps over 444.5 s. Both implementations are in Python with C++ sections for the computing-intensive portions. The further addition of OpenMP multiprocessing during the C++ computation of the normalized deviations and derivatives reduced the LBFGS runtime to 322 s (64 cores, accelerating equations 10, 11 and 15).

## 4. Discussion   

The phasing of serial crystallographic data has been notoriously difficult. Unlike in the rotation method, where the integration, scaling, error-treatment and merging protocols have been well studied, in SX the algorithms continue to be refined to account for the sparseness of the data recorded from each crystal. Most of the few *de novo* XFEL structures in the PDB required tens to hundreds of thousands of images to solve (Barends *et al.*, 2014 ▸; Nakane *et al.*, 2015 ▸, 2016 ▸; Colletier *et al.*, 2016 ▸; Nass *et al.*, 2016 ▸; Hunter *et al.*, 2016 ▸; Yamashita *et al.*, 2015 ▸; Gorel *et al.*, 2017 ▸). In this work, we have shown that some of the difficulty arises from how the error estimates are treated and used when merging SX data. In addition to affecting the final merged intensity by being used in the weighted sum, the merged error estimates themselves are used extensively in the maximum-likelihood techniques used by phasing algorithms. For example, in McCoy *et al.* (2004 ▸), equation (2) describes the probability of the magnitudes of a set of unphased structure factors given a set of phased structure-factor vectors. While this equation uses only the intensities as inputs, a set of adjustments propagated from the estimated experimental error is presented in Appendix *B* [App appb] as initial values used in maximum-likelihood refinement. With this, it is not surprising that accurate estimates of the errors are useful, but it is notable here how striking the difference improving the estimates of errors in the measured reflections makes in the ability to phase XFEL data.

It also interesting to note how important using a good set of weights is when merging the data using a weighted mean. Protocol 1 (unweighted mean) performed consistently better than protocol 2 (weighted mean), even though a weighted mean should be a better estimator of a population mean (especially for the left-skewed intensity distributions seen in XFEL data). Stated differently, using the photon-counting error estimates alone as weights is not optimal, at least in terms of this anomalous phasing exercise. It is only after adjusting the individual measurement error estimates such that they better explain the observed variance through the Ev11 approach that using the error estimates as weights improves the results over the unweighted mean (protocol 3, Ev11).

By no means do we assert that the methods presented here are an exhaustive list of possible ways of treating the errors in XFEL data collection. While we want to note that using the initial estimates of error from integration and applying adjustments to bring them closer to explaining the observed error is important for *de novo* phasing success, at least when using a weighted sum for averaging intensities together, none of the methods here propagate the error from the partiality correction itself. From (1)–(3) we see that the intensities are corrected using a partiality term, a scale factor and a Wilson *B* factor. (2) propagates the error in inflating *I_hj_* by *K_hj_*, assuming that *K_hj_* is a constant, but in reality the parameters comprising *K_hj_* are refined quantities. The partiality is dependent on the crystal orientation, unit-cell dimensions, wavelength spectrum and estimated mosaicity (Sauter, 2015 ▸), and while the true errors in these terms are unknown, they could be estimated from the population of crystals used for merging and then propagated to the factor *K_hj_* (see Appendix *B* [App appb]). Likewise, the estimated error estimates of these terms could be refined. Initial efforts in this direction have been made and can be accessed using experimental parameters in *cctbx.xfel*. While this still will not account for the full set of unknown random and systematic errors present in SX data collection, any error propagation in this manner should reduce the reliance on inflationary terms to account for the observed variance in the sample.

## 5. Software availability   

Instructions for downloading and using *cctbx.xfel* are available at the cctbx.xfel wiki at http://cci.lbl.gov/xfel. See also Brewster *et al.* (2019 ▸) for instructions on using the *cctbx.xfel* graphical user interface (GUI).

## Figures and Tables

**Table 1:** Summary of error-modeling methods

Protocol	Weight†	Description
1	—	Unweighted error estimates
2	σ_*hj*_	Photon-counting error estimates as weights
3	σ_Ev11_	Refine SDFAC terms to inflate photon-counting error estimates

† These are the weights used in (7) and (8), such that the weight *w* = 1/σ^2^.

**Table 2:** SAD phasing results for different error models Values in parentheses are for the highest resolution bin.

Protocol	1	2	3
Weight†	—	σ_*hj*_	σ_Ev11_
Resolution (Å)	80.78–1.80 (1.86–1.80)		
*I*/σ‡	13.8 (2.7)	59.7 (2.0)	14.0 (1.4)
CC_1/2_ (%)	99.9 (73.8)	99.8 (63.3)	99.9 (81.4)
Zn^2+^ peak height (σ)	53.1	50.5	67.0
No. of sites found by *HySS* §	6 ± 0	6 ± 0	6 ± 0
No. of residues built (of 316)§	252.4 ± 15.2	104.1 ± 1.4	297.2 ± 6.5
Model–map CC§ ¶ (%)	71.0 ± 0.4	30.3 ± 0.2	80.0 ± 0.1
*R*_work_ § (%)	27.4 ± 1.8	54.8 ± 0.3	21.2 ± 1.3
*R*_free_ § (%)	29.9 ± 2.0	57.3 ± 0.8	23.7 ± 1.7

† As in Table 1 ▸, for a given weight *w* where *w* = 1/σ^2^.

‡ These are higher than in Fig. 3 ▸ because the higher intensity observations are given a higher weight during merging.

§ Numbers are mean ± standard deviation over ten trials with differing random-number seeds.

¶ Phased map correlation to the known structure.

## Footnotes

1 These terms are re-expressed from the Sdfac, SdB and Sdadd terms in Evans (2011 ▸), such that *s*_fac_ = Sdfac, *s*_B_ = (SdB)^1/2^ and *s*_add_ = Sdadd.

## Appendix A Derivatives of the target function

As part of least-squares minimization we take the partial derivative of (14) with respect to each of the *s*_fac_, *s*_B_ and *s*_add_ parameters, which we refer collectively here as the parameters *p*. We can perform this via (10), (13), (14) and the chain rule. We first take derivatives with respect to the square of each parameter:

Since the intensity value as used in the computation of δ_*bk*norm_ does not depend on the parameters being refined,

We can now compute the partial derivatives of (10) with respect to the parameters *p*. Note that the minimizer refines the terms themselves instead of the squares of the terms, and that (∂*p* ^2^/∂*p*) = 2*p*. Therefore,

## Appendix B Error propagation for partial reflections

In this work, for each reflection we compute a scaling term *K*_*hj*_ that includes the partiality correction, the Wilson *B* factor and the scaling factor *G*. *K*_*hj*_ depends on the crystal orientation, unit-cell parameters, wavelength, mosaicity and so forth (equations 1 and 3). A simple error propagation including the photon-counting error σ^P^ *_hj_* and assuming no error in *K*_*hj*_ is shown in (2). However, if estimates of the errors in terms comprising *K*_*hj*_ were available they could be propagated, and the first few steps of this process are shown here. Given parameters *p* (*p*_1_, *p*_2_, …) that contribute to *K*_*hj*_, the propagated error is

where again σ^P^ *_hj_* is the photon-counting error and σ^2^ *_hj_* is the propagated error. By the chain rule,

which reduces to (2) if the errors in the parameters *p* are ignored.

Initial implementation of these and further derivatives of *K*_*hj*_ with respect to the parameters *p* as well as refinement of the associated error terms is available through experimental options in *cctbx.xfel*.
